# One‐End‐Truncated Au_52_ and Au_70_ Quantum Rods: High‐Quantum‐Yield NIR‐II Emitters With Aspect‐Ratio‐Dominated Photophysics

**DOI:** 10.1002/anie.1815171

**Published:** 2026-09-01

**Authors:** Lianshun Luo, Avirup Sardar, Guiying He, Abhrojyoti Mazumder, Guoxiang Hu, Gangli Wang, Rongchao Jin

**Affiliations:** ^1^ Department of Chemistry Carnegie Mellon University Pittsburgh Pennsylvania USA; ^2^ School of Chemistry and Biochemistry Georgia Institute of Technology Atlanta Georgia USA; ^3^ Department of Chemistry Georgia State University Atlanta Georgia USA

**Keywords:** atomically precise nanoclusters, luminescence, near‐infrared‐II, periodicity, quantum rods

## Abstract

Precise control over absorption/emission in NIR‐II (1000–1700 nm) is essential for advancing photonic technologies. Atomically precise gold quantum rods (QRs) provide a structurally well‐defined platform for fine‐tuning NIR‐II anisotropic excitonic behavior at the atomic level. In this study, we report Au_52_(PET)_38_ and Au_70_(PET)_50_ (PET = 2‐phenylethanethiolate) as members of a distinct branch of Au QRs with one‐end‐truncated kernel topology. The Au_52_ and Au_70_ exhibit NIR‐II absorption at 1032 and 1275 nm and nanosecond fluorescence at 1100 and 1360 nm, respectively. Their high quantum yields (20.9% for Au_52_ and 14.8% for Au_70_) together with high brightness (5.6 × 10^4^ M^−1^ cm^−1^ for Au_52_ and a similar order of magnitude for Au_70_) substantially exceed those of most molecular NIR‐II dyes, highlighting their promise as efficient inorganic NIR‐II fluorophores. Comparative analysis across two structurally distinct Au QR families, including one‐end‐truncated and double‐pointed rods, reveals that the longitudinal absorption and emission peak wavelengths, as well as the fluorescence linewidths, follow a common aspect‐ratio‐dependent scaling relation, suggesting length‐dependent modulation of the ground‐ and excited‐state electronic structure. These findings uncover a hierarchical interplay between rod elongation and kernel topology in defining NIR‐II excitonic behavior and expand the structural design space of atomically precise QRs.

## Graphical abstract

Two nanoclusters, Au_52_(PET)_38_ and Au_70_(PET)_50_, where PET = 2‐phenylethanethiolate, are isolated, which constitute a series of one‐end‐truncated kernel topologies. Both Au_52_ and Au_70_ exhibit NIR‐II absorption at 1032 and 1275 nm and nanosecond fluorescence at 1100 and 1360 nm, respectively, with high quantum yields (20.9% for Au_52_ and 14.8% for Au_70_) and high brightness (of order 10^4^ M^−1^ cm^−1^).

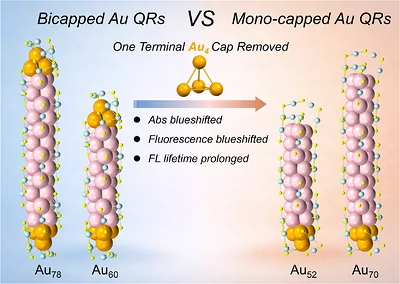

Atomically precise metal nanoclusters (NCs) have recently gained wide attention for applications in optoelectronics, photonics, and catalysis [1, 2, 3, 4, 5]. Photoluminescence in the second near‐infrared (NIR‐II, ∼1000–1700 nm) has emerged as a rapidly expanding frontier in optics, energy, chirality, and bio‐related technologies. Relative to the visible (400–700 nm) and NIR‐I light (700–900 nm), NIR‐II photons possess advantages of reduced scattering, low‐loss transmission, high atmospheric transparency, and minimal background interference, enabling deeper penetration and enhanced signal fidelity. In addition to these advantages, NIR‐II constitutes a substantial fraction of the solar spectrum (∼20%–25%) [6], underscoring the broader relevance for energy‐related applications [7].

Atomically precise NCs are a versatile class of NIR‐II‐responsive materials due to their exceptional photophysical properties and atomic‐level tunability [7, 8, 9, 10, 11, 12]. They display tunable absorption and emission across the entire NIR‐II window, positioning them among the most spectrally extensive NIR‐II materials reported to date [13, 14, 15, 16, 17, 18]. Furthermore, their ultrasmall size, intrinsic photostability, and chemically modifiable surfaces make them well suited for biocompatible probe design and translational biomedical imaging [19, 20, 21]. Most importantly, control over the structure [7], core size [22, 23, 24], shape [25, 26, 27], chirality [5, 28, 29], composition [30, 31, 32], and ligand environment [33, 34, 35, 36], allows for deliberate single‐atom‐level “surgery”, affording an unprecedented programmable level of control over the optical responses and an understanding of structure‐property relationships at the atomic level.

The recent success in synthesizing atomically precise gold quantum rods (abbrev. Au QRs) [37, 38, 39, 40, 41], pioneered by our group, has unveiled a new structural evolution that gives rise to unprecedented optical behaviors. These QRs originate from Au_24_(PET)_20_ [42], which elongates by a periodic addition of a Au_18_(PET)_12_ unit (PET = 2‐phenylethanethiolate) along one dimension (1D), forming Au_42_(PET)_32_, Au_60_(PET)_44_, Au_78_(PET)_56_, Au_96_(PET)_68_, and Au_114_(PET)_80_ QRs with aspect ratio (AR) ranging from 6.3 to 18.7 [40]. Such a growth pattern is further supported by thermally induced 1D oligomerization of Au_24_(PET)_20_ into the QR series by the Tsukuda group [43]. The 1D elongation gives rise to strongly polarized longitudinal excitonic transitions [37, 40, 44, 45] and exceptionally intense absorption with broad tunability from ∼800 to ∼1730 nm (peak) [40]. Our group recently reported that the Au_60_, Au_78_, Au_96_, and Au_114_ QRs exhibit ultrabright, sub‐nanosecond fluorescence spanning the wavelength range of 1000–2200 nm (edge to edge), thus, covering both NIR‐II and shortwave infrared (SWIR, 1700–2500 nm) regions, with brightness values (*γ*∼10^4−^ ^5^M^−1^ cm^−1^) surpassing state‐of‐the‐art NIR‐II organic dyes by two orders of magnitude and SWIR dyes by more than four orders of magnitude [46], revealing unprecedented photophysical performance of these atomically precise rod‐shaped NCs.

In this study, we report Au_52_(PET)_38_ and Au_70_(PET)_50_ QRs with pronounced absorption and strong fluorescence in the NIR‐II. Compared to the reported Au_24_‐derived series of QRs, both Au_52_ and Au_70_ preserve the three‐atom‐wide cross section but they do not originate from the double‐pointed Au_24_(PET)_20_, rather, they originate from Au_34_(PET)_26_ that features a one‐end‐truncated kernel topology. The elongation from Au_34_(PET)_26_ to Au_52_(PET)_38_ to Au_70_(PET)_50_ follows the same periodic Au_18_(PET)_12_ addition along the longitudinal axis. The Au_52_ and Au_70_ display NIR‐II longitudinal absorption peaks at 1032 and 1275 nm, with emission maxima at 1100 nm (quantum yield (QY) ∼20.9%) and 1360 nm (QY∼14.8%), respectively. Furthermore, the high molar extinction coefficient (*ε*) of Au_52_ at 1032 nm, 2.66 × 10^5^ M^−1^ cm^−1^, offers a high brightness (*γ*) of 5.6 × 10^4^ M^−1^ cm^−1^. We further reveal how the kernel end‐topology influences other emission behavior of Au QRs. This work broadens the structural diversity of Au QRs and deepens our understanding of structure‐enabled control in atomically precise 1D NCs.

The synthesis of the Au QRs follows an NHC‐mediated method (NHC = N‐heterocyclic carbene) developed by our group [38, 47, 48]. The product was purified by thin‐layer chromatography (TLC). As shown in Figures 1A and S1, distinct bands are clearly resolved on the TLC plate. From the top (elution fastest) to the bottom (slowest), the Au_52_ and Au_70_ species appear as relatively faint bands compared to their slightly larger neighbors (Au_60_ and Au_78_ reported before [40]), indicating their lower abundances (or yields) in the crude product mixture. It is worth noting that Tsukuda's group recently observed the same series via a thermally induced 1D oligomerization of Au_24_(PET)_20_ [43], in which Au_52_ and Au_70_ were also formed in low yields, but no pure product was isolated [43]. Thus, our current work is the first successful isolation of pure Au_52_ and Au_70_ species for optical studies. In this bottom‐up route, the molecular NHC–Au(I) precursor may influence the speciation of Au(I)–SR intermediates and the kinetics of reduction‐induced nucleation and anisotropic growth. By contrast, thermal oligomerization proceeds through the fusion and structural reconstruction of preformed Au_24_(PET)_20_ NCs. Together, these distinct but convergent pathways provide complementary strategies for generating Au QRs.

**FIGURE 1:**
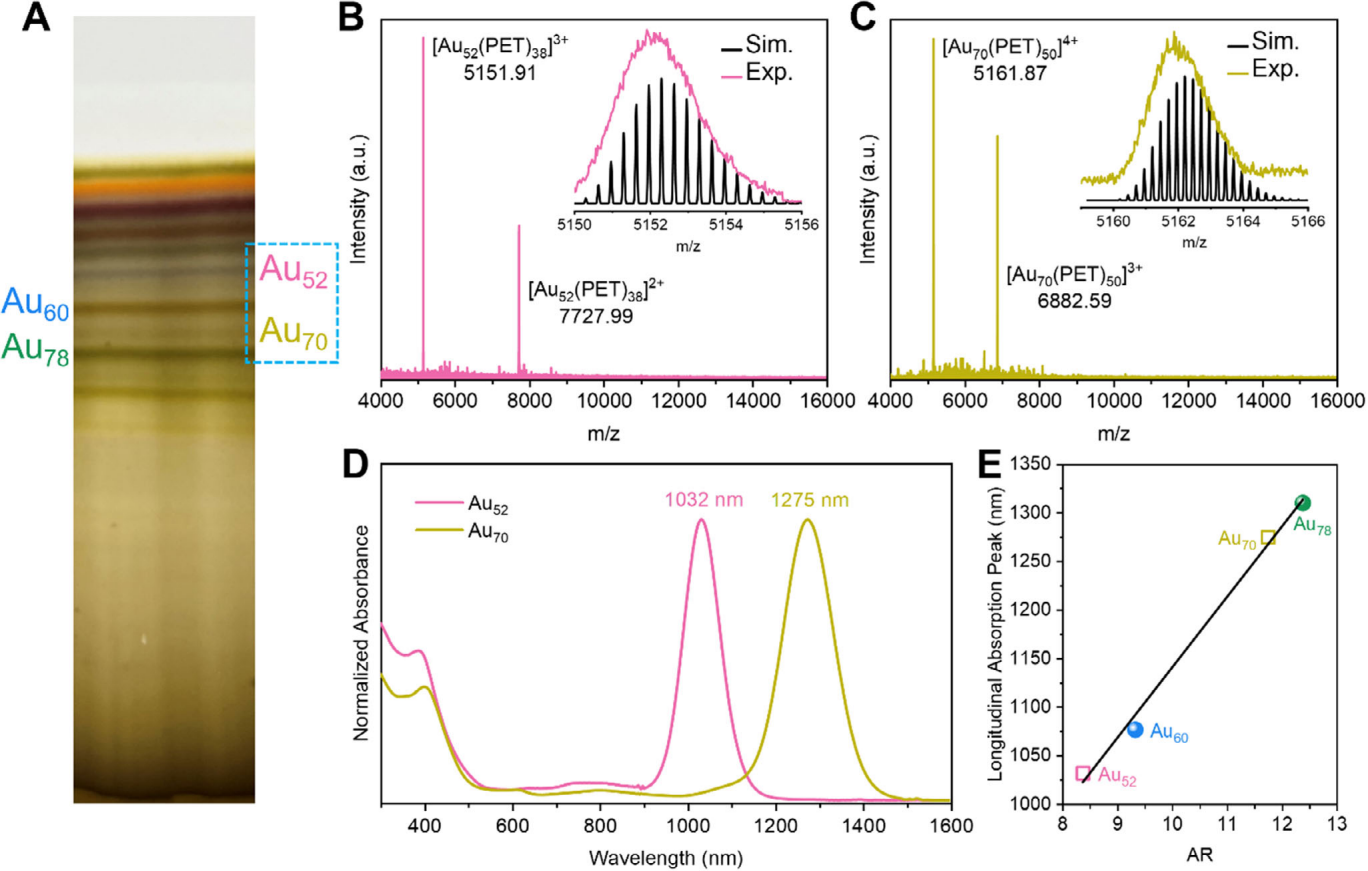
(A) TLC separation of Au_52_(PET)_38_, Au_60_(PET)_44_, Au_70_(PET)_50_, and Au_78_(PET)_56_ from the product mixture. (B) ESI mass spectrum of Au_52_(PET)_38_; inset shows the experimental isotope pattern of [Au_52_(PET)_38_]^3+^ (*m*/*z* = 5151.91) and the simulated pattern (black). (C) ESI mass spectrum of Au_70_(PET)_50_; inset shows the experimental isotope pattern of [Au_70_(PET)_50_]^4+^ (*m*/*z* = 5161.87) and the simulated pattern (black). The approximately 1 Da deviation in the deconvoluted molecular mass corresponds to only 65 and 48 ppm for Au_52_ and Au_70_, respectively, and is within the mass accuracy of the Q‐TOF measurements in the high‐*m/z* region. (D) Normalized optical absorption spectra of Au_52_ and Au_70_ QRs (dissolved in toluene‐d_8_ to avoid overtone vibrational peaks in the NIR‐II range). (E) Longitudinal absorption peak wavelength plotted against AR for Au_52_, Au_60_, Au_70_, and Au_78_ QRs.

Electrospray ionization mass spectrometry (ESI‐MS) analyses of Au_52_ and Au_70_ show peaks corresponding to [Au_52_(PET)_38_]^3+^ and [Au_70_(PET)_50_]^4+^ (Figures 1B,C). Although cesium acetate was used as an ionization additive, no Cs‐containing adducts were detected. These gas‐phase charge states most likely arise from mass spectrometric in‐source ionization rather than the native charges of the NCs. We have also considered the superatom electron counts using $N=n_{\text{Au}}-m_{\text{SR}}-q$. The 3+ and 2+ charge states of Au_52_(PET)_38_ formally correspond to 11 and 12 electrons, respectively, whereas the 4+ and 3+ charge states of Au_70_(PET)_50_ correspond to 16 and 17 electrons, respectively. Among these charge states, only [Au_52_(PET)_38_]^2+^ has a shell‐closing electron count of 12 according to the prolate jellium model. Therefore, the overall ESI charge‐state distributions cannot be explained solely by electronic shell closure and should not be interpreted as direct evidence of the native charge states of the QRs in solution. The experimental isotope patterns match well with the calculated patterns, confirming the deduced formulas of Au_52_(PET)_38_ and Au_70_(PET)_50_. The optical absorption spectra of purified Au_52_ and Au_70_ are shown in Figure 1D. Both Au_52_ and Au_70_ exhibit distinct NIR‐II absorption peaks centered at 1032 and 1275 nm, respectively, together with a common absorption peak at ∼400 nm; Note: the latter peak is nearly constant for all the QRs [37, 38, 39, 40, 41, 43, 49]. Such optical behaviors of Au QRs (note: the energy gap *E*_g_ = 1.05 eV for Au_52_ and 0.85 eV for Au_70_, Figure S2) arise from the quantized electronic structure (i.e., excitons upon photoexcitation), as opposed to the metallic/plasmonic nanorods [50, 51]. The exciton assignment is further supported by our previous DFT and TD‐DFT calculations on the structurally homologous (vide infra) Au_60_ and Au_78_ QRs [40]. The molar extinction coefficient (*ε*) of Au_52_ at 1032 nm was determined to be 2.66 × 10^5^ M^−1^ cm^−1^ (Figure S3), slightly lower than that of Au_60_ (2.97 × 10^5^ M^−1^ cm^−1^) [40]. The limited yield of Au_70_ precluded a reliable determination of its *ε*; however, its structural and spectral similarities to Au_78_ suggest a value on the order of 10^5^ M^−1^ cm^−1^. Moreover, the optical gaps of Au_52_ and Au_70_ follow the same one‐dimensional particle‐in‐a‐box relation as the previously reported double‐end Au QRs (Figure S2C) [40], confirming a common quantum‐confinement behavior. The spectral profiles of Au_52_ and Au_70_ closely resemble those of previously reported Au_60_ and Au_78_ (Figure S4), indicating their structural relationship (vide infra). Moreover, the absorption maxima progressively redshift from Au_52_ to Au_78_. Importantly, when the longitudinal absorption peak wavelengths of Au_52_, Au_60_, Au_70_, and Au_78_ are plotted against the rods‘ ARs, a clear linear correlation emerges (Figure 1E), demonstrating that the longitudinal absorption follows a universal AR‐dependent scaling law across both QR series, regardless of the kernel end‐topology. All of these results indicate the analogous anisotropy‐driven electronic transitions and support a stepwise structural evolution within the Au_52_–Au_70_ QR series.

The structural evolution from Au_24_ to Au_42_ and further to Au_60_, Au_78_, Au_96_, and Au_114_ was established in our previous studies [40], involving the sequential addition of one to five Au_18_(PET)_12_ units to the three‐atom‐wide, prolate Au_8_ kernel (i.e., Au_4_–Au_4_) in Au_24_. The kernel evolution of Au QRs follows Au_4_–(Au_3_)_4_ *_n_*–Au_4_ (where *n* denotes the number of Au_18_(PET)_12_ units, *n* = 1 to 5), whereas the overall elongation can be generalized by the formula Au_24+18_ *_n_* (PET)_20+12_ *_n_* (Figure 2A, entry 1). This relationship reflects a periodic elongation along the longitudinal axis of the QRs, with Au_24_(PET)_20_ serving as the beginning of the evolution. As illustrated in Figure 2B (left), sequential addition of two Au_18_(PET)_12_ units to Au_24_ gives rise to Au_60_, and adding three units produces Au_78_, accompanied by an increase in AR from 9.3 to 12.4. Below, we rationalize the structural growth of Au_52_ and Au_70_ QRs.

**FIGURE 2:**
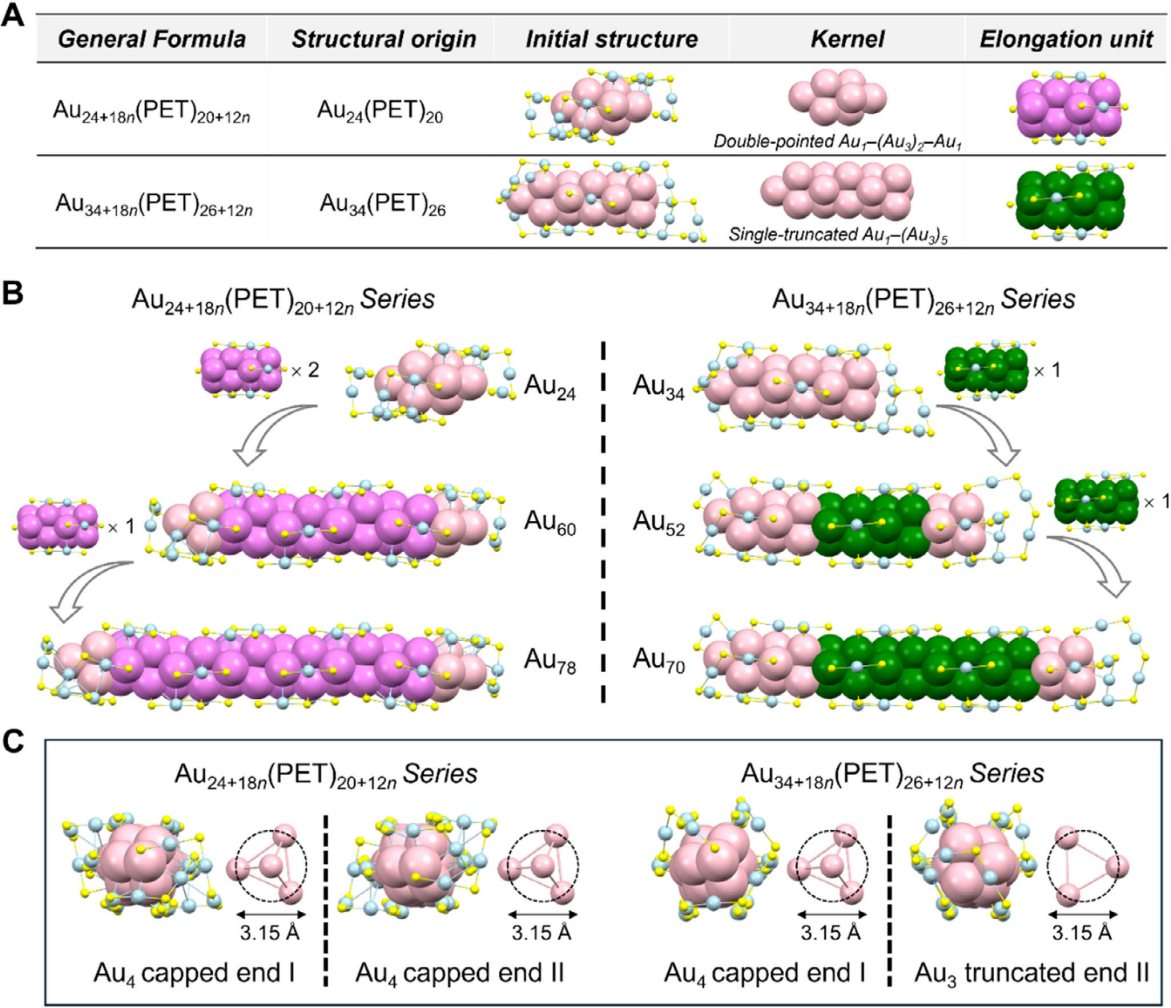
(A) Generalized construction schemes of two series of Au QRs. (B) Structural evolution pathways of Au_24+18_ *_n_* (PET)_20+12_ *_n_* and Au_34+18_ *_n_* (PET)_26+12_ *_n_* series. (C) End‐on views along the longitudinal axis of the double‐end (left two views) and one‐end‐truncated (right two views) Au QR series.

A recent report of Au_34_(SR)_26_ with a kernel structure of Au_4_−(Au_3_)_4_ by Tsukuda's group [41] reveals an interesting relation with the Au_42_(SR)_32_ QR [37] that possesses a kernel of Au_4_−(Au_3_)_4_−Au_4_, that is, one Au_4_‐end is truncated in the formation of Au_34_(SR)_26_ and the formulas differ by Au_8_(SR)_6_. This trend is also present in the pair of Au_52_(PET)_38_ and Au_60_(PET)_44_, as well as the pair of Au_70_(PET)_50_ and Au_78_(PET)_56_, implying a similar Au_4_‐end truncation. Together with their common absorption peak at ∼400 nm and resemblant long‐wavelength peaks, we deduce the structures of Au_52_ and Au_70_ as similar rod structures that were determined by x‐ray crystallography [37, 40, 41]. While Au_52_ and Au_70_ do not follow the Au_24+18_ *_n_* (PET)_20+12_ *_n_* formula series, rather, the Au_34+18_ *_n_* (PET)_26+12_ *_n_* formula series (Figure 2A, entry 2) [43], the same Au_18_(PET)_12_ increment is adopted in the growth from Au_34_ to Au_52_ to Au_70_, suggesting that Au_52_ and Au_70_ follow an analogous Au_18_(PET)_12_‐based elongation, with the structural starting point being Au_34_ (as opposed to Au_24_). Based on the Au_34_(SR)_26_ x‐ray structure and analysis reported by Tsukuda et al. [41], Au_52_ and Au_70_ can be assigned to a distinct QR branch that follows the general formula Au_34+18_ *_n_* (PET)_26+12_ *_n_* (Figure 2A). In this series, the structural evolution originates from Au_34_(PET)_26_, subsequent elongation proceeds through the periodic addition of Au_18_(PET)_12_ unit along the longitudinal axis, giving rise to Au_52_ and Au_70_ as shown in Figure 2B (right). Accordingly, the Au kernels in this new series follow the general expression Au_4_–(Au_3_)_4_ *_n_* (monocapped), distinguishing them from the bicapped Au_4_–(Au_3_)_4_ *_n_*–Au_4_ series [40, 43]. The Au_52_ and Au_70_ have ARs of 8.4 and 11.7, respectively, which are slightly smaller than those of Au_60_ (AR = 9.3) and Au_78_ (AR = 12.4) due to one Au_4_‐end truncation. While we attempted to isolate and purify Au_88_(PET)_62_ through repeated TLC separations, its very low initial abundance in the crude product and the sample loss during cumulative purification (requiring multiple runs for high purity) prevented us from obtaining any pure sample. Thus, the absence of Au_88_(PET)_62_ from the present study reflects the practical difficulty of its isolation and purification, rather than the termination of this QR series at *n* = 2. Further optimization of the synthesis for higher yields and better separation will be required to characterize the Au_88_(PET)_62_ and even longer QRs in this series.

Both QR series (Au_24_ to Au_114_ vs. Au_34_ to Au_70_) adopt Au_18_(PET)_12_ as the common elongation unit but differ in their surface architectures. The longitudinal and end‐on views (Figures 2B,C) show that both series retain the same three‐atom‐wide *hcp* kernel cross section and transverse kernel diameter of 3.15 Å. However, they differ in terminal Au_4_ capping and in the spatial distributions of the Au–thiolate motifs over the rod surfaces. Therefore, while the Au_18_(PET)_12_‐based elongation mechanism is preserved across both series, their ends’ topology and surface motif distribution are different, establishing Au_52_ and Au_70_ as new members of a surface‐structure‐different yet correlated QR branch of the previously reported Au_24_ to Au_114_ series [40, 43].

As discussed above, all four Au QRs (Au_52_, Au_60_, Au_70_, and Au_78_) are from the NaBH_4_‐mediated synthesis [40] and share an identical three‐atom‐wide cross section while exhibiting progressively increasing lengths. However, they exhibit distinct topologies: Au_52_ and Au_70_ feature mono‐truncated kernels, whereas Au_60_ and Au_78_ retain the bi‐capped feature. The end topology difference is manifested in the optical absorption by peak shifts (see Figure S4). We next investigate the PL properties of Au_52_ and Au_70_. Both Au_52_ and Au_70_ display pronounced NIR‐II emission (Figure 3A), with peaks centered at 1100 and 1360 nm, respectively. Notably, both spectra are blue‐shifted by ∼45 nm relative to their counterparts Au_60_ and Au_78_ (Figure S5). Furthermore, Au_52_ exhibits a high QY of 20.9% (Figure S6) and Au_70_ exhibits a QY of 14.8% (Figure S7), respectively, comparable to those of Au_60_ and Au_78_. The Au_60_ QR exhibits NIR‐II emission centered at 1145 nm with a QY of 20.9%, and elongation to Au_78_ results in a redshifted emission at 1400 nm with a QY of 15.8% [46]. Combined with the measured *ε*, the QY of Au_52_ gives a brightness of 5.6 × 10^4^ M^−1^ cm^−1^, comparable to that of Au_60_ (6.2 × 10^4^ M^−1^ cm^−1^). Although the low isolated yield of Au_70_ precluded an experimental determination of its brightness, its close structural and photophysical similarity to Au_78_ hints a brightness in the 10^4–5^ M^−1^ cm^−1^ range. The PL lifetime is 1.4 ns (Figure 3B, for emission at 1100 nm) for Au_52_ and 1.0 ns (Figure 3C, for emission at 1360 nm) for Au_70_, confirming the fluorescence nature. These fluorescence lifetimes are slightly longer than those of Au_60_ (0.9 ns) and Au_78_ (0.8 ns) [46], which should be caused by the end topology difference of the two types of QRs.

**FIGURE 3:**
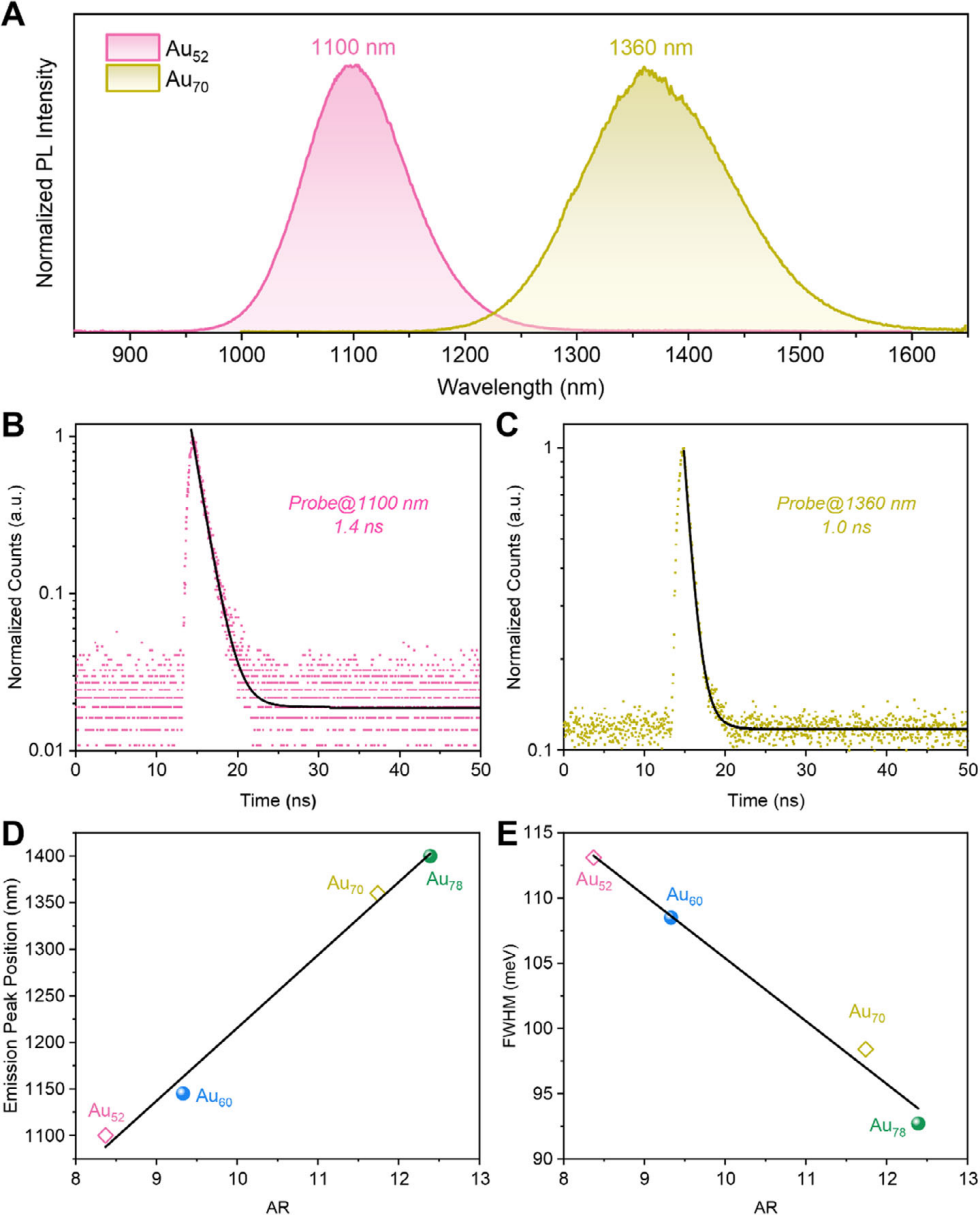
(A) Normalized PL spectra of Au QRs in toluene‐d_8_. For excitation: 1032 nm for Au_52_ and 1275 nm for Au_70_. (B) Decay profile of the 1100 nm emission peak of Au_52_ QR. (C) Decay profile of the 1360 nm emission peak of Au_70_ QR (excitation: ∼80 ps pulsed laser of 375 nm wavelength, instrument response function (IRF) ∼400 ps). (D) The emission peak position versus the AR. (E) The FWHM of PL versus the AR.

Within the series of Au_24+18_ *_n_* (PET)_20+12_ *_n_*, the emission peaks of Au_60_, Au_78_, Au_96_, and Au_114_ exhibit an excellent linear correlation with AR (Figure S8). Such a linear dependence demonstrates that the radiative transition (the peak wavelength) scales linearly with rod elongation. This trend parallels the previously established AR‐dependent scaling of the longitudinal absorption in the QR series, underscoring a common AR‐governed scaling of the exciton behavior [50, 51]. The Au_52_ and Au_70_ possess the same three‐atom‐wide cross section but an end‐truncated kernel topology. Consequently, their ARs (i.e., 8.4 for Au_52_ and 11.7 for Au_70_) are slightly lower than those of Au_60_ (AR = 9.3) and Au_78_ (AR = 12.4). When plotted against AR (Figure 3D), the emission peaks of Au_52_ and Au_70_ fall on the same linear trend defined by the Au_24+18_ *_n_* (PET)_20+12_ *_n_* series. This result indicates that, despite their different surface motifs and end topologies, the radiative transition wavelengths of both series obey the same AR‐dependent scaling relationship. Therefore, the emission wavelength of Au QRs is primarily dictated by longitudinal extension, while the effects of the surface motifs and end‐topology are minor.

Beyond the emission peak position, we also examine the emission linewidth (FWHM, in meV) as a function of AR, as it provides complementary insight into how rod elongation and kernel topology influence the excitons. Within the series of Au_24+18_ *_n_* (PET)_20+12_ *_n_*, the FWHM decreases linearly with increasing AR (Figure S9). This systematic narrowing indicates a length‐determined evolution of the emissive excitonic state. The linear FWHM‐AR relationship is consistent with reduced longitudinal quantum confinement and corresponding contraction of energy level spacing upon elongation. For Au_52_ and Au_70_, the FWHM likewise decreases with increasing AR (Figure 3E), indicating that longitudinal elongation remains the dominant factor for controlling the excitonic linewidth evolution. Furthermore, their linewidths largely collapse onto the linear FWHM–AR relation defined by the Au_24+18_ *_n_* (PET)_20+12_ *_n_* series (Figure 3E). Overall, these observations suggest that rod elongation (i.e., AR) is the primary structural parameter dictating the emission linewidth in Au QRs, whereas differences in kernel topology introduce minor perturbations.

We further compare the photophysical properties of Au_52_ and Au_70_ with relevant NCs reported in the literature (Table 1). Both Au_52_ and Au_70_ exhibit pronounced NIR‐II optical features, with absorption at 1032 and 1275 nm and emission at 1100 and 1360 nm, respectively. In contrast, previously reported Au_52_ NCs with the face‐centered cubic (fcc), tetra‐icosahedral, or bi‐icosahedral kernels typically show absorption peaks in the shorter wavelengths (≤ 920 nm) and emission maxima ≤ 980 nm. The markedly red‐shifted optical response of Au_52_ and Au_70_ into NIR‐II therefore highlights the unique photophysical characteristics of the rod‐like hcp kernels. This behavior originates from the anisotropic 1D elongation of the three‐atom‐wide Au rod kernel, which generates a strongly polarized longitudinal excitonic transition and leads to intense NIR‐II absorption and emission. The pronounced influence of atomic‐level structural configuration on NIR photophysics has also been demonstrated in geometrically distinct bi‐icosahedral Au NCs [56]. Variations in kernel geometry can reorganize the electronic levels and coupling, whereas the ligand environments can modify surface rigidity and excited‐state relaxation [13, 35, 36, 52]. By contrast, longitudinal extension within the Au_52_/Au_70_ series provides a systematic, AR‐dependent control over NIR‐II absorption and emission while retaining the three‐atom‐wide hcp kernel, PET ligand packing, and one‐end‐truncated topology.

**TABLE 1:** Summary of photophysical properties of Au_52_ and Au_70_ and a comparison with iso‐sized NCs.

Nanoclusters	Kernel type	Abs peak (nm)	Em peak (nm)	QY (%)	PL lifetime	References
**Au_52_(PET)_38_**	**hcp rod**	**1032**	**1100**	**20.9**	**1.4 ns**	**This work**
Au_52_(PET)_32_	fcc rod	770	915	3.2	8.07 µs	[49]
Au_52_(*p*‐MBT)_32_	fcc rod	790	899	18.3	554 ns	[52]
Au_52_(4‐EBT)_32_	fcc rod	790	916	14.7	465 ns	
Au_52_(IPBT)_32_	fcc rod	790	922	11.1	322 ns	
Au_52_(TBBT)_32_	fcc rod	790	938	3.8	134 ns	
Au_52_(HOPPh_2_)_8_(OPPh_2_)_4_(TBBT)_16_	tetra‐icosahedral	920	—	—	—	[53]
Au_20_Ag_32_(*^t^* BuPA)_24_Cl_12_	bi‐icosahedral	835	980	33	1.85 µs	[54]
**Au_70_(PET)_50_**	**hcp rod**	**1275**	**1360**	**14.8**	**1.0 ns**	**This work**
Au_70_S_20_(PPh_3_)_12_	fcc	—	—	—	—	[55]

In summary, two Au QRs (Au_52_ and Au_70_) are obtained in this work, and their structure and photophysical properties are studied. In contrast to the Au_24+18_ *_n_* (PET)_20+12_ *_n_* periodic series (including Au_24_, Au_42_, Au_60_, Au_78_, Au_96_, and Au_114_), Au_52_ and Au_70_ retain the same three‐atom‐wide rod cross section but adopt one end‐truncated kernel topology and evolve from an Au_34_‐based origin through a periodic Au_18_(PET)_12_ elongation. Both Au_52_ and Au_70_ exhibit pronounced longitudinal absorption in the NIR‐II region, with maxima centered at 1032 and 1275 nm, respectively, accompanied by strong NIR‐II fluorescence at 1100 nm for Au_52_ and 1360 nm for Au_70_. Importantly, their longitudinal absorption and emission peak wavelengths, as well as the fluorescence linewidths, follow the same AR‐dependent scaling that is established for the Au_24+18_ *_n_* (PET)_20+12_ *_n_* series, demonstrating that the longitudinal elongation, rather than the capping Au_4_ ends, governs the excitonic evolution and emission broadening of Au QRs. This work expands the structural landscape of atomically precise Au QRs, and the findings deepen our understanding of how precise atomic structure governs NIR‐II photophysics.

## Author Contributions

**Lianshun Luo**: investigation and writing – original draft. **Avirup Sardar**: investigation. **Guiying He**: investigation. **Abhrojyoti Mazumder**: investigation. **Guoxiang Hu**: investigation. **Gangli Wang**: investigation. **Rongchao Jin**: investigation and writing – review and editing.

## Conflicts of Interest

The authors declare no conflicts of interest.

## Supporting information




**Supporting File**: anie74531‐sup‐0001‐SuppMat.pdf.

## Data Availability

The data that support the findings of this study are available in the Supporting Information of this article.
