# Supplementary material for: One‐End‐Truncated Au52 and Au70 Quantum Rods: High‐Quantum‐Yield NIR‐II Emitters With Aspect‐Ratio‐Dominated Photophysics
Source: Angew Chem Int Ed Engl. 2026 Sep 1;65(42):e1815171. doi: 10.1002/anie.1815171 (PMC13653617; doi:10.1002/anie.1815171)
Supplement: Supplementary file 1 [file ANIE-65-e1815171-s001.pdf]

Chemicals

Tetrachloroauric (III) acid ($HAuCl_4 \cdot 3H_2O$, 99.999% metal basis, Aldrich), phenylethyl mercaptan (PET, >99%, Aldrich), sodium borohydride (powder, ≥98%, Sigma), benzimidazole (98%, Aldrich), 2-bromopropane (99%, Aldrich), dimethyl sulfide (SMe_2, >99%, Aldrich), potassium carbonate (K_2CO_3, 99%, Aldrich), toluene-d8 (99 atom % D, Sigma), deuterium oxide (D_2O, 99.9 atom % D, Sigma), tetrachloroethylene (C_2Cl_4), methanol, ethanol, ethyl ether, ethyl acetate, pentane, dichloromethane (DCM), chloroform, *n*-hexane and acetonitrile (HPLC grade for all solvents) were used as received. Thin-layer chromatography (TLC) plates were from iChromatography (silica gel, 250 μm).

Synthesis of chloro(dimethylsulfide)gold(I) (AuCl(SMe$_2$))

$HAuCl_4 \cdot 3H_2O$ (500 mg, 1.27 mmol) was dissolved in ethanol (20 mL), followed by the addition of SMe_2 (280 μL, 3.81 mmol), and the solution was vigorously stirred for 2 hours. After that, the white precipitate was collected by centrifugation, which was washed with ethyl ether and finally dried to give the product as a white powder.

Synthesis of 1,3-diisopropylbenzimidazolium bromide (iPr$_2$-bimy·HBr)

The synthesis of iPr$_2$-bimy·HBr was performed according to a previously reported procedure with minor modifications.[1] In a typical procedure, benzimidazole (1.18 g, 10 mmol) and K_2CO_3 (760 mg, 5.5 mmol) were added into acetonitrile (8 mL) and the mixture was rapidly stirred at ambient temperature for 1 hour. Following that, 2-bromopropane (5.4 mL, 57.5 mmol) was added to the suspension, and the reaction mixture was vigorously stirred under reflux conditions for 24 hours, followed by the addition of a second portion of 2-bromopropane (5.4 mL, 57.5 mmol). The reaction mixture was vigorously stirred under reflux for an additional 48 hours. After removing the solvent under reduced pressure, DCM was added to the residues, and the upper supernatant after centrifugation was collected. The solvent of the supernatant was removed under reduced pressure to produce a spongy solid, which was washed by ethyl acetate to afford the desired product as a white powder.

Synthesis of NHC-Au-Br complex (^{i}Pr$_2$-bimy·AuBr)

The synthesis of ^{i}Pr$_2$-bimy·AuBr was performed according to a previously reported procedure with minor modifications.[2] ^{i}Pr$_2$-bimy·HBr (1337.4 mg, 4.725 mmol), AuCl(SMe$_2$) (1393.4 mg, 4.725 mmol), and K_2CO_3 (653.5 mg, 4.725 mmol) were added into acetone (20 mL) and the mixture was vigorously stirred under reflux conditions for 2 hours. After that, the solvent in the suspension was removed under reduced pressure. DCM was added to the residues, and the upper supernatant after centrifugation was collected. The solvent of the supernatant was removed under reduced pressure to give the solid product, which was washed with pentane and finally dried to afford the desired product as a gray powder.

Synthesis of Au quantum rods

^{i}Pr$_2$-bimy·AuBr (120 mg, 0.25 mmol) and PET (67 μL, 0.5 mmol) were dissolved in a mixture

of chloroform (15 mL) and ethanol (5 mL). The mixture gradually turned cloudy white (Au^{I}-PET formed). After 1 hour of stirring, the suspension was reduced to nanoclusters (NCs) by the addition of sodium borohydride (95 mg, 2.5 mmol, dissolved in 5 mL ice-cold H_2O) under darkness. A black solution was formed. The reaction was allowed to continue for 6 hours, and then the solvent was removed under reduced pressure. The mixture of Au NCs was thoroughly washed with methanol, extracted with DCM, and concentrated for TLC separation. The mixture of Au NCs was pipetted onto a TLC plate, and the separation was conducted in a developing tank (developing solvent 1:1 (v/v) DCM:*n*-hexane). Four bands corresponding to $Au_{52}(PET)_{38}$, $Au_{60}(PET)_{44}$, $Au_{70}(PET)_{50}$, and $Au_{78}(PET)_{56}$ were cut off (**Figure S1**) and dissolved in DCM for characterization.

Electrospray ionization mass spectrometry (ESI-MS)

ESI-MS were performed on a Waters Q-TOF mass spectrometer equipped with Z-spray source. The sample was dissolved in dichloromethane and diluted (2:1 v) by dry methanol containing 50 mM cesium acetate (≥99.99%, Aldrich). The source temperature was kept at 70 °C. The sample was directly infused into the chamber at 5 μL/min. The spray voltage was kept at 2.20 kV and the cone voltage at 60 V.

Steady-state UV-Vis-NIR measurements

UV-Vis-NIR spectra of the nanoclusters were collected with a UV-3600 Plus spectrophotometer (Shimadzu, detection range: 185 to 3300 nm).

Steady-state photoluminescence and lifetime measurements

Steady-state photoluminescence (PL) spectra were recorded using an FLS-1000 spectrofluorometer (Edinburgh Instruments). Emission (up to ~1650 nm) was measured using a wide-range photomultiplier tube (PMT-1700, cooled to –80 °C with liquid nitrogen).

The PL lifetimes were measured by time-correlated single photon counting (TCSPC) with the PMT-1700 detector (up to ~1650 nm) on the same instrument. The excitation was ~80 ps pulsed laser (~375 nm).

Quantum yield measurement

The absolute QYs (PL up to 1650 nm with the PMT-1700 detector) were measured using an integrating sphere, the standard sample holder was removed and replaced with the integrating sphere. First, a blank spectrum was recorded by placing a cuvette containing only the solvent in the integrating sphere and performing five repeated emission scans, starting from 20 nm longer than the excitation wavelength to the longest wavelength of the detector. Next, the blank was replaced with a dilute solution of Au QRs adjusted to an OD of 0.1 at the excitation wavelength. The emission scan was then repeated five times over the same spectral range. PLQY was determined using the software of FLS-1000.

The relative quantum yields (Φ_S) of other samples (denoted S in the equation below) beyond the coverage by the PMT-1700 detector were measured by using a reference standard (denoted R in the equation) under the same instrumental settings and calculated using the following equation:

$$\Phi_S = \Phi_R(\frac{I_S}{I_R})(\frac{1-10^{-A_R}}{1-10^{-A_S}})(\frac{n_S}{n_R})^2$$

where, Φ_R is the QY of the reference standard, *I* is the integrated PL intensity (for S or R), *A* is the absorbance of the solution at the excitation wavelength (for S and R), and *n* is the refractive index of the solution solvent.

Supporting Figures:

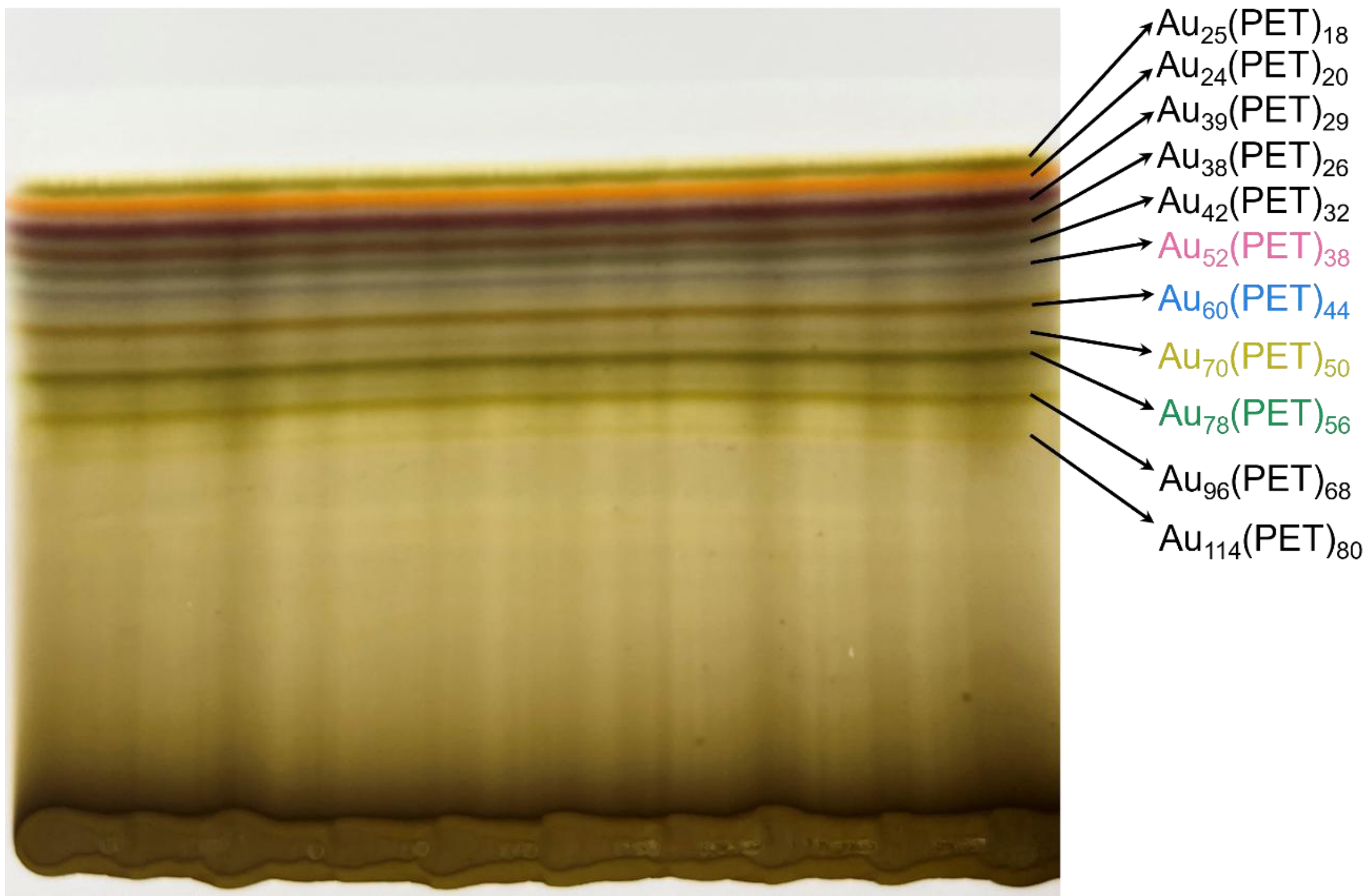

Figure S1. TLC separation of $Au_{52}(PET)_{38}$, $Au_{60}(PET)_{44}$, $Au_{70}(PET)_{50}$, and $Au_{78}(PET)_{56}$, from the product mixture.

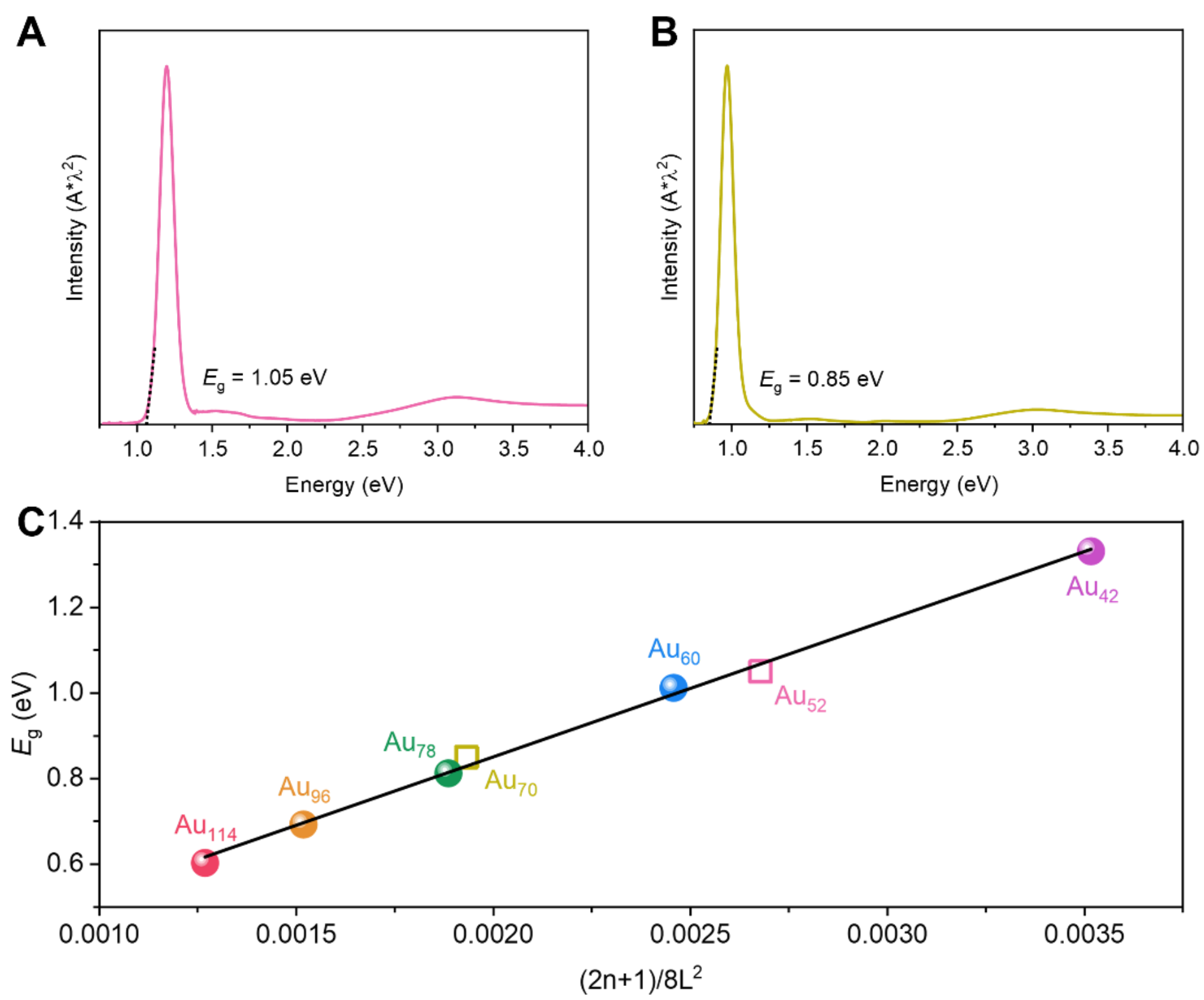

Figure S2. Absorption spectrum on photon energy scale (absorbance×λ^2 versus photon energy) of (A) $Au_{52}(PET)_{38}$ and (B) $Au_{70}(PET)_{50}$, both HOMO-LUMO gaps are determined by extrapolation to zero absorbance). (C) Fitting of optical gaps of the two series of QRs by the Schrödinger equation for the 1D particle-in-a-box; *L*: the length of Au QRs, *n*: the electronic energy level.

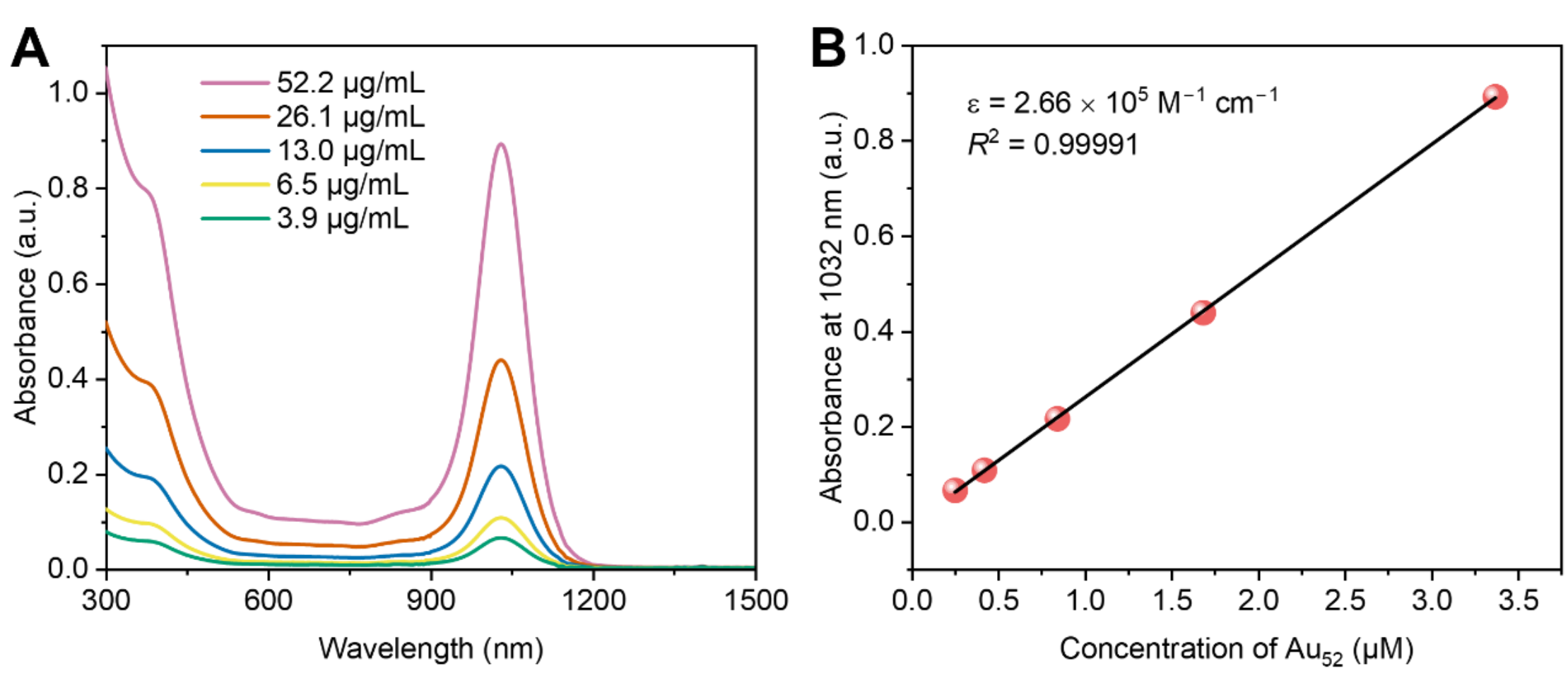

Figure S3. (A) Absorption spectra of Au_{52} with different concentrations in toluene-d_8. (B) Absorbance of Au_{52} at 1032 nm vs. concentration and the linear fit, the absorption coefficient at 1032 nm $\varepsilon = 2.66 \times 10^5$ M^{-1} cm^{-1}.

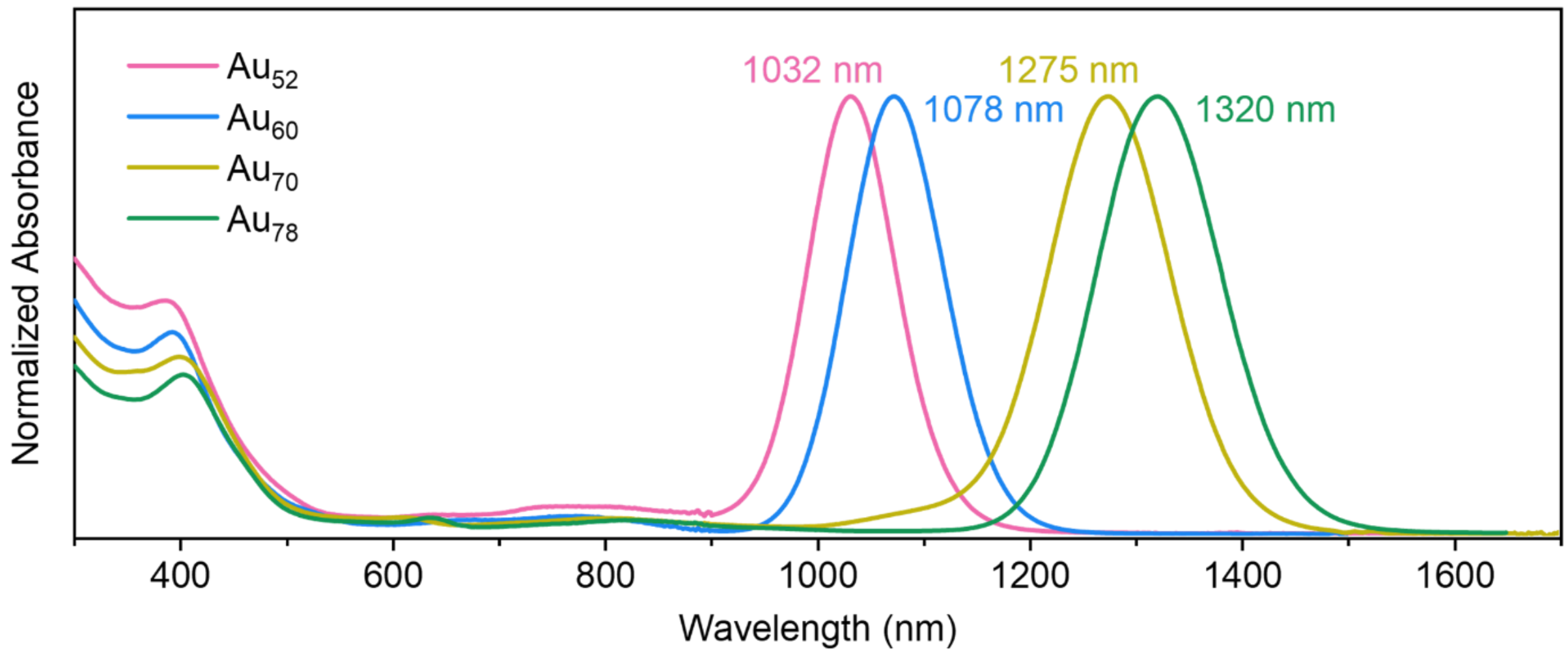

Figure S4. Normalized optical absorption spectra of Au QRs (dissolved in toluene-d_8 to avoid overtone vibrational peaks in the NIR-II range).

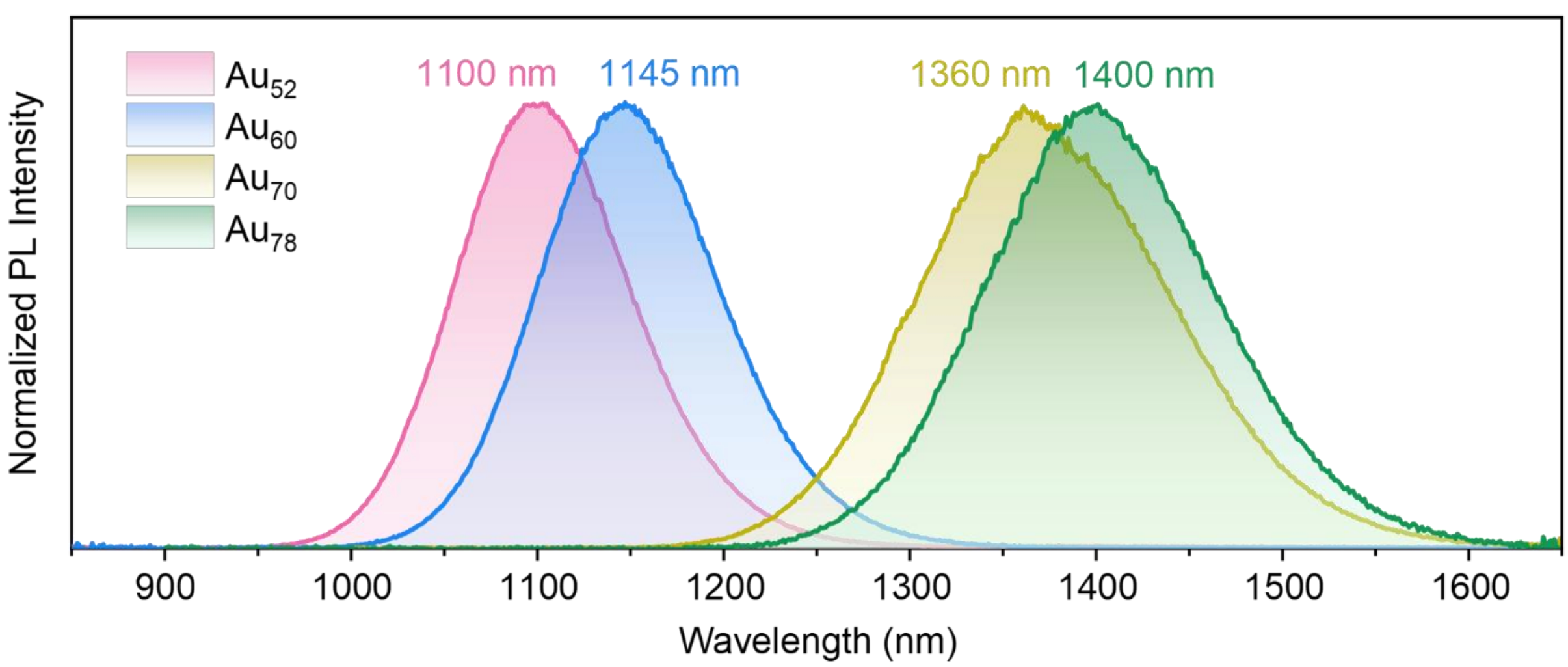

Figure S5. Normalized PL spectra of Au QRs in toluene-d_8. For excitation: 1032 nm for Au_{52}, 1078 nm for Au_{60}, 1275 nm for Au_{70}, and 1320 nm for Au_{78}.

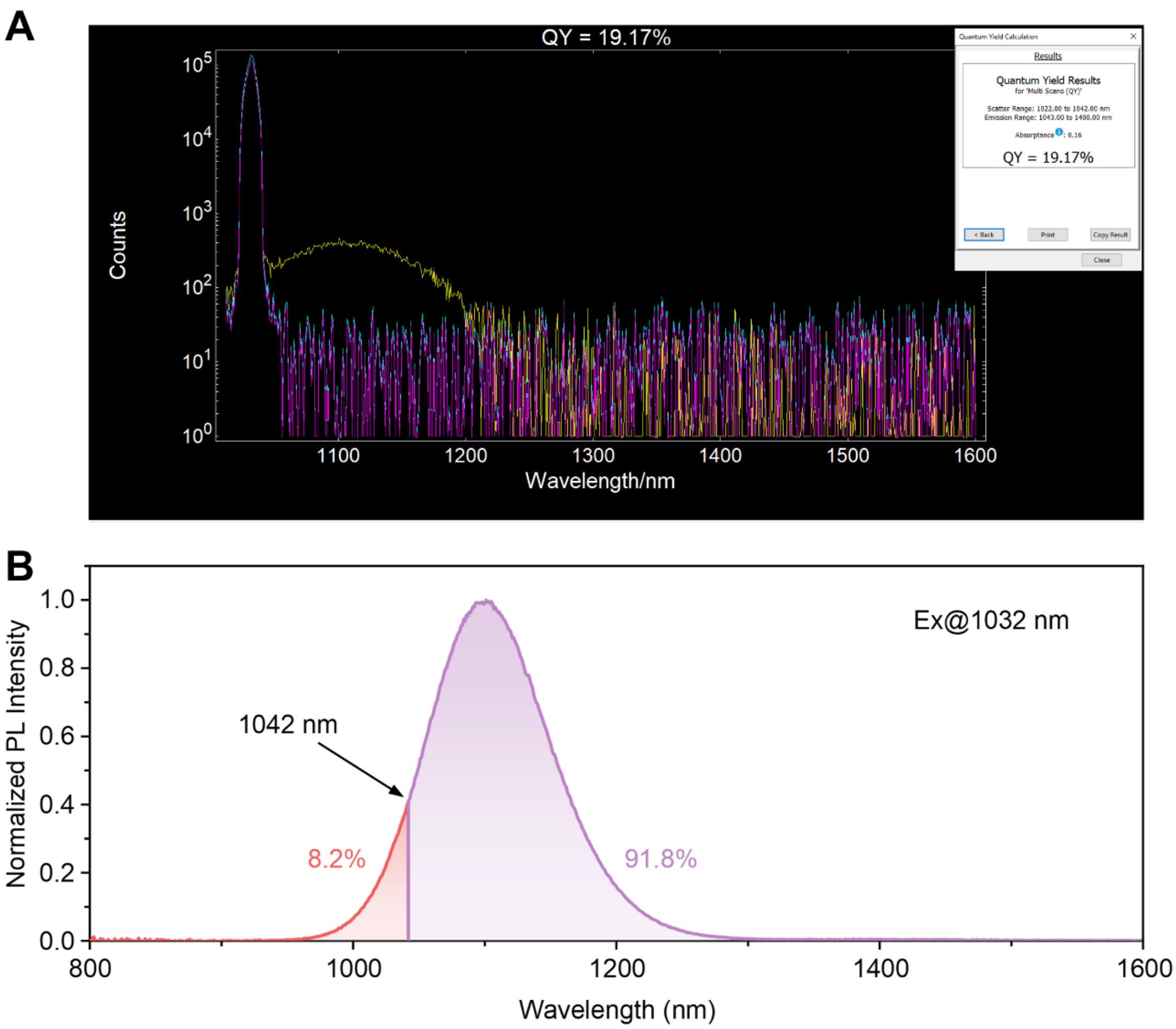

Figure S6. (A) QY measurement of Au_{52} in toluene-d_8 under the excitation of 1032 nm, corresponding to the NIR-II absorption peak of Au_{52}. Excitation slit bandwidth was set to 10 nm. (B) PL spectrum of Au_{52} in toluene-d_8 under 1032 nm excitation, showing the integrated PL intensity contributions from 800-1042 nm (8.2% of the total PL intensity, i.e. area) and 1042-1600 nm (91.8% of the total PL intensity).

The excitation wavelength was set to 1032 nm with an excitation slit bandwidth of 10 nm. As a result, the system integrates the emission signal from 1042 to 1600 nm, excluding the portion between 800 and 1042 nm. Therefore, the measured QY value (19.17% in **Figure S6A**) represents only the emission >1042 nm. To obtain the accurate total QY of Au_{52} under 1032 nm excitation, the emission between 800 and 1042 nm should be included (8.2% of the total PL intensity in **Figure S6B**), yielding a corrected QY of $\frac{1}{91.8\%} * 19.17\% = 20.9\%$.

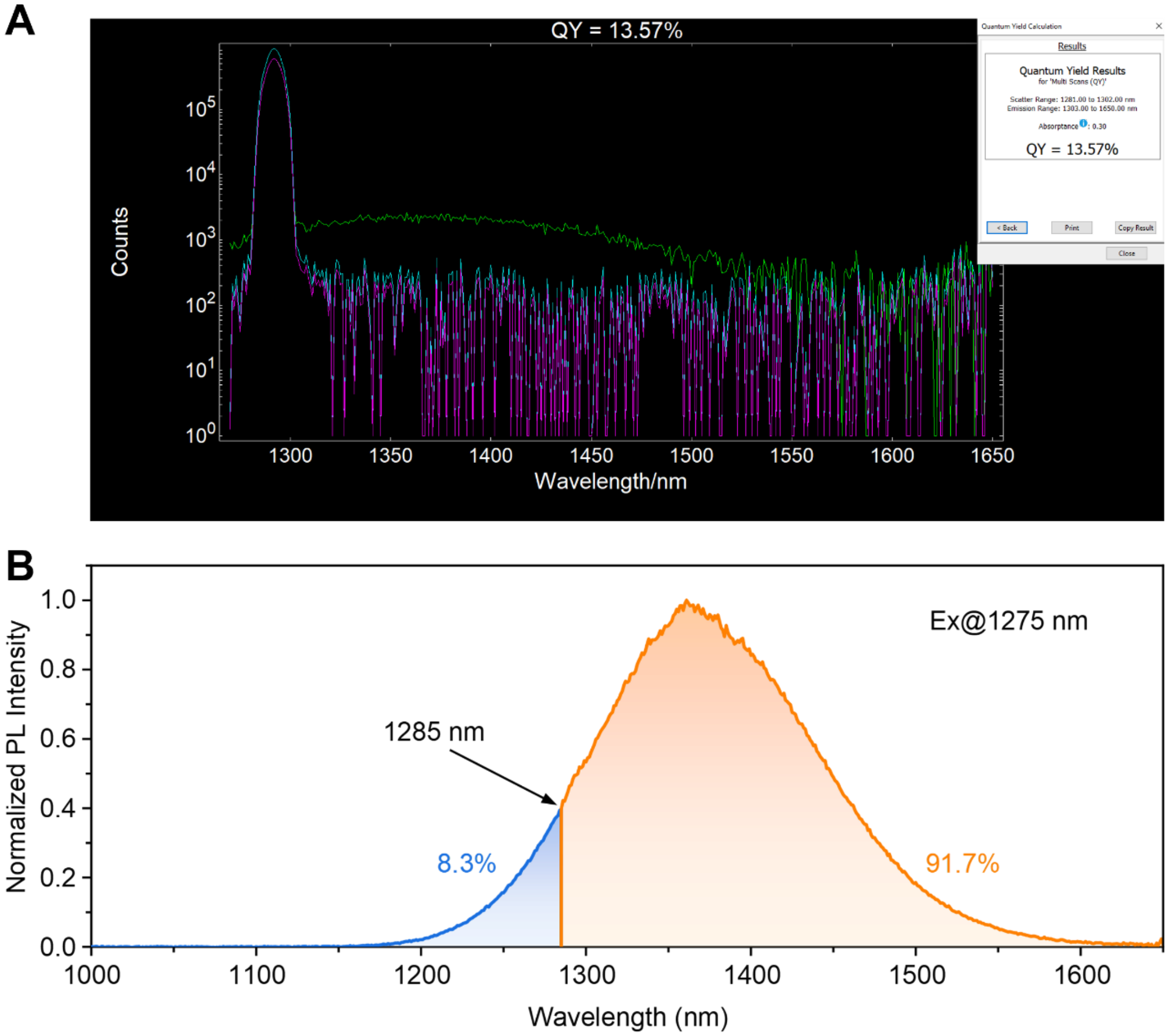

Figure S7. (A) QY measurement of Au_{70} in toluene-d_8 under the excitation of 1275 nm, corresponding to the NIR-II absorption peak of Au_{70}. Excitation slit bandwidth was set to 10 nm. (B) PL spectrum of Au_{70} in toluene-d_8 under 1275 nm excitation, showing the integrated PL intensity contributions from 1000-1285 nm (8.3% of the total PL intensity, i.e. area) and 1285-1650 nm (91.7% of the total PL intensity).

The excitation wavelength was set to 1275 nm with an excitation slit bandwidth of 10 nm. As a result, the system integrates the emission signal from 1285 to 1650 nm, excluding the portion between 1000 and 1285 nm. Therefore, the measured QY value (13.57% in **Figure S7A**) represents only the emission >1285 nm. To obtain the accurate total QY of Au_{70} under 1275 nm excitation, the emission between 1000 and 1285 nm should be included (8.3% of the total PL intensity in **Figure S7B**), yielding a corrected QY of $\frac{1}{91.7\%} * 13.57\% = 14.8\%$.

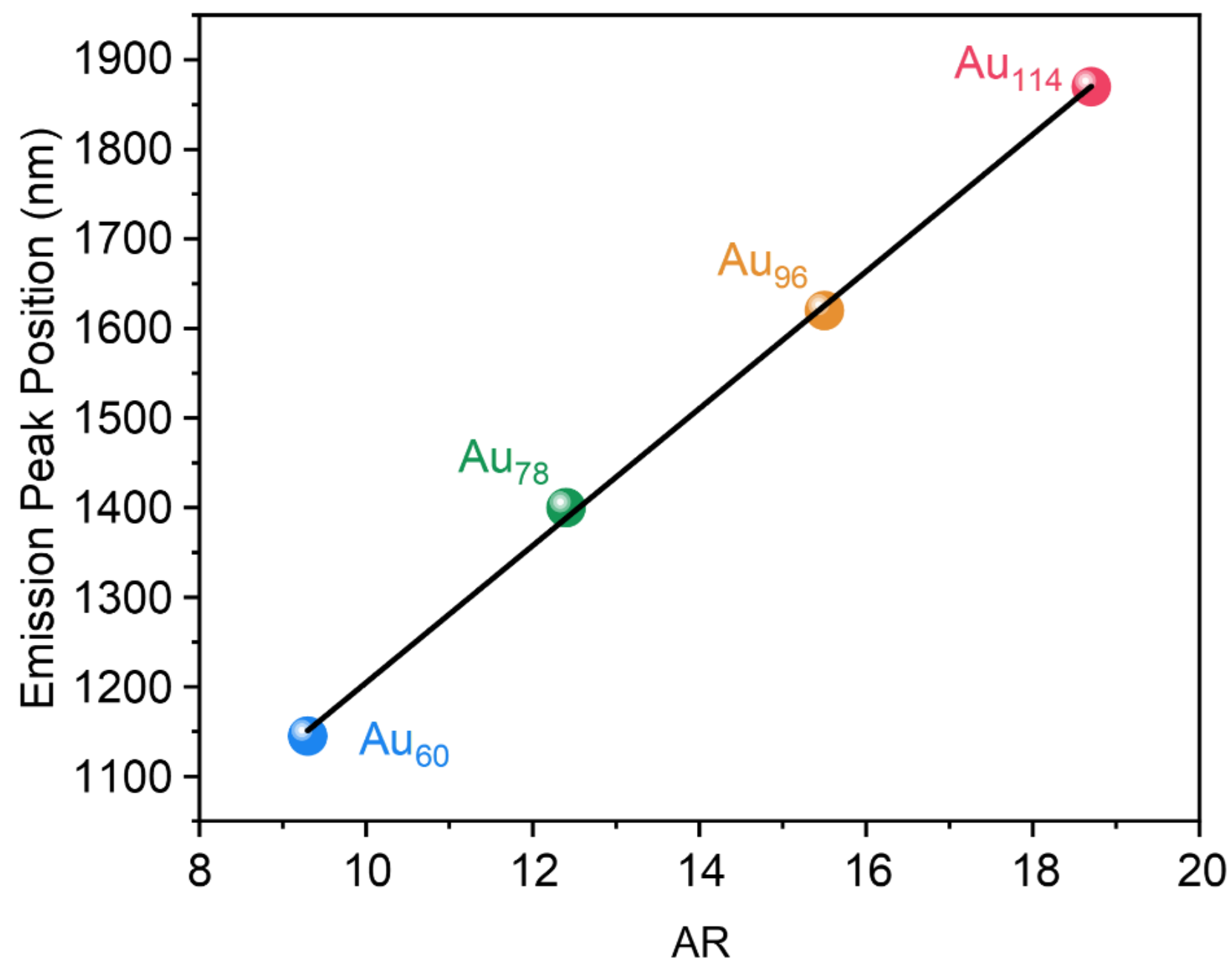

Figure S8. The PL emission peak position vs. the AR.

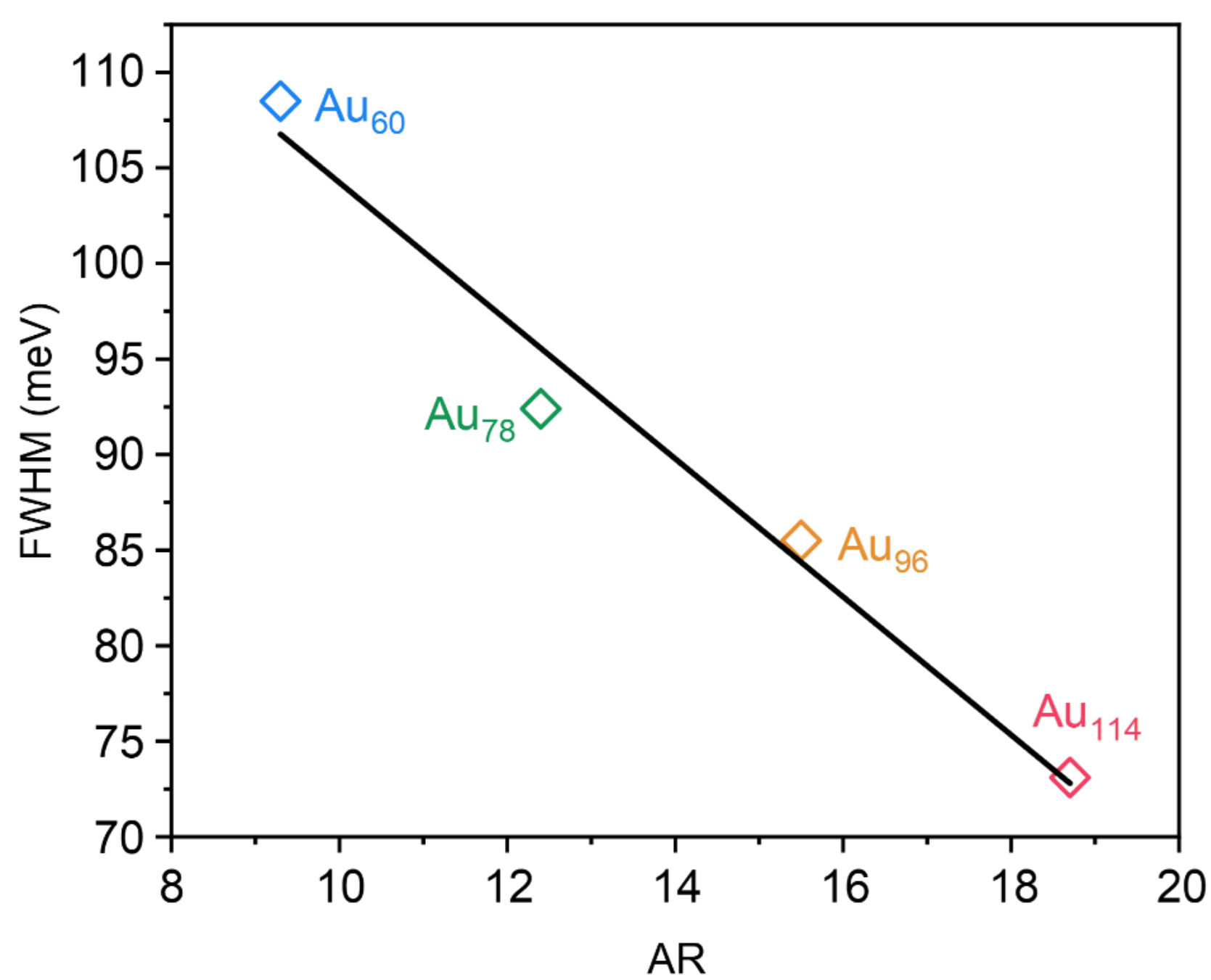

Figure S9. The FWHM of PL vs. the AR.

Supporting references

1. Huynh, H. V.; Han, Y.; Ho, J. H. H.; Tan, G. K. Palladium-(II) complexes of a sterically bulky, benzannulated N-heterocyclic carbene with unusual intramolecular C–H···Pd and $C_{carbene}$···Br interactions and their catalytic activities. *Organometallics* **2006**, 25, 3267-3274.
2. Luo, L.; Liu, Z.; Du, X.; Jin, R. Photoluminescence of the $Au_{38}(SR)_{26}$ Nanocluster Comprises Three Radiative Processes. *Commun. Chem.* **2023**, *6*, 22.
